# Shedding Light on Extracellular Vesicle Biogenesis and Bioengineering

**DOI:** 10.1002/advs.202003505

**Published:** 2020-11-27

**Authors:** Fei Teng, Martin Fussenegger

**Affiliations:** ^1^ Department of Biosystems Science and Engineering ETH Zurich Mattenstrasse 26 Basel CH‐4058 Switzerland; ^2^ Faculty of Science University of Basel Mattenstrasse 26 Basel CH‐4058 Switzerland

**Keywords:** bioengineering, delivery systems, exosomes, extracellular vesicles, microvesicles

## Abstract

Extracellular vesicles (EVs) are biocompatible, nano‐sized secreted vesicles containing many types of biomolecules, including proteins, RNAs, DNAs, lipids, and metabolites. Their low immunogenicity and ability to functionally modify recipient cells by transferring diverse bioactive constituents make them an excellent candidate for a next‐generation drug delivery system. Here, the recent advances in EV biology and emerging strategies of EV bioengineering are summarized, and the prospects for clinical translation of bioengineered EVs and the challenges to be overcome are discussed.

## Graphical abstract

Extracellular vesicles (EVs) managing diverse intercellular communication networks are attracting increasing interest in developing next‐generation drug delivery systems. This review provides a comprehensive state‐of‐the‐art coverage of EV biogenesis, transport, release, and uptake as well as cargo sorting and discusses the progress and challenges of engineering EVs for therapeutic applications.

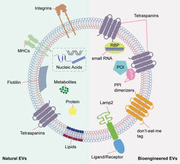

## 1 Introduction

Extracellular vesicles (EVs) are heterogeneous nano‐sized membrane vesicles that are released from various cell types.^[^ ^1^ ^]^ Classically, there are three major subtypes of EVs defined on the basis of their biological pathways, i.e., exosomes, microvesicles, and apoptotic bodies.^[^ ^1^, ^2^, ^3^, ^4^, ^5^, ^6^, ^7^ ^]^ Exosomes are vesicles formed by inward invagination of the endosomal membrane and fusion of the multivesicular body (MVB) with the plasma membrane. Microvesicles are formed by direct outward budding from the plasma membrane of healthy cells. Apoptotic bodies are formed during apoptosis by outward blebbing of the plasma membrane of cells. With the advance of EV studies, diverse EV subtypes have been proposed, using terms such as ectosomes, microparticles, oncosomes, etc., in addition to the three major types mentioned above.^[^ ^7^, ^8^ ^]^ The different terminologies based on function, biogenesis, size or cell of origin, as well as the variety of isolation methods and contexts, and the paucity of validated biomarkers, have led to various misconceptions and even contradictory definitions in the literature.^[^ ^7^, ^10^ ^]^ In this context, the International Society for Extracellular Vesicles (ISEV) has proposed the term “extracellular vesicle” as generic nomenclature for cell‐released vesicles, and has also recommended minimal requirements for EV studies.^[^ ^7^, ^11^ ^]^ In addition, the EV‐TRACK platform has been launched to facilitate standardization of EV research through more systematic reporting of EV biology and methodology.^[^ ^9^ ^]^

In this review, we will use EVs as a generic term for the entire population of vesicles secreted from cells, since most research described in the literature has utilized heterogeneous populations of EVs, and often failed to characterize the isolated EVs in detail.^[^ ^7,8^ ^]^ EVs were originally discovered by Clargaff and West in 1946 as procoagulant platelet‐derived particles in normal plasma.^[^ ^12^ ^]^ Since then, the biology and functions of EVs have been widely investigated (for example, see refs. [13, 14] reviewed in refs. [1, 15]). In 1983, EVs derived from fusion of MVBs with the plasma membrane of reticulocytes were observed.^[^ ^16^, ^17^ ^]^ Subsequently, EVs were implicated in immune regulation^[^ ^18^, ^19^ ^]^ and transfer of genetic materials,^[^ ^20^, ^21^, ^22^ ^]^ highlighting their role in cell–cell communication. It has been established that EVs contain thousands of different biomolecules, including proteins, RNAs, DNAs, lipids, and metabolites,^[^ ^5^, ^23^, ^24^ ^]^ and are highly heterogeneous in terms of their size, content, functional impacts on recipient cells, and cell of origin.^[^ ^15^, ^25^ ^]^ Here, we focus mainly on the most extensively studied EVs, typically designated as exosomes and microvesicles (ectosomes), which play key roles in intercellular communication by delivering signals to recipient cells.^[^ ^26^, ^27^ ^]^ These EVs hold great promise for developing next‐generation delivery vehicles for therapeutic agents.

## 2 EV Biogenesis and Secretion

**Figure 1:**
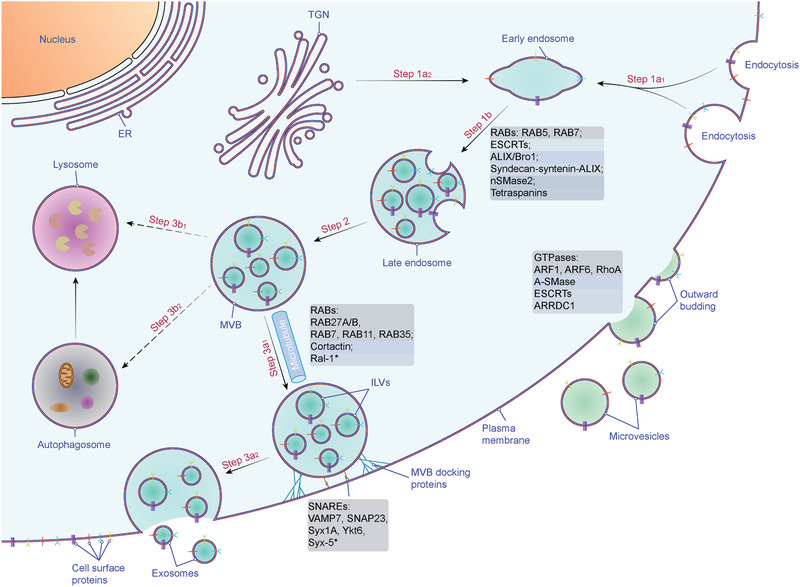
Biogenesis and secretion of EVs. Two mechanisms of EV biogenesis are illustrated. The process of releasing exosomes into the extracellular milieu contains three distinct steps: exosome biogenesis, intracellular trafficking of MVBs, and fusion of MVBs with the plasma membrane. Early endosomes are formed by the inward budding of the plasma membrane (Step 1a_1_), or in some cases from the *trans*‐Golgi network (TGN) (Step 1a_2_). Early endosomes mature into late endosomes (Step 1b) and finally generate MVBs, in which process ILVs are formed by inward invagination of the endosome limiting membrane (Step 2). The fate of MVBs can be fusion with the plasma membrane (Step 3a_1_), which results in the release of exosomes (Step 3a_2_). Alternatively, MVBs can fuse with lysosomes/autophagosomes for degradation (Step 3b_1, 2_). Several molecules are involved in the biogenesis (e.g., RABs, ESCRTs, syndecan, ceramide, tetraspanins, etc.), trafficking (e.g., RABs, actin, etc.), and fusion of MVBs with the plasma membrane (e.g., SNAREs). Microvesicles arise from the direct outward budding and fission of the plasma membrane. Several molecules are involved in the biogenesis and release of microvesicles (small GTPases, ESCRTs, ARRDC1, *etc*.). Abbreviations: EV, extracellular vesicle; ESCRT, endosome sorting complex required for transport; MVB, multivesicular body; ILV, intraluminal vesicle; RAB, RAS‐related protein; ALIX, ALG‐2 interacting protein X; nSMase2, neutral sphingomyelinase 2; Ral‐1, RAL (Ras‐related GTPase) homolog; SNARE, soluble NSF attachment protein receptor; VAMP7, vesicle‐associated membrane protein 7; SNAP23, synaptosomal‐associated protein 23; Syx1A, syntaxin 1A; ARF, ADP ribosylation factor; RohA, Ras homolog family member A; A‐SMase, acid sphingomyelinase; ARRDC1, arrestin domain containing protein 1. *, homologs in *C. elegans*.

EVs are formed through multiple mechanisms (**Figure** **1**). Classically, exosome biogenesis is initiated from the endosomal pathway, i.e., the formation of early endosomes via invagination of the plasma membrane. In some cases, vesicles derived from the budding of the *trans*‐Golgi network (TGN) can fuse with early endosomes.^[^ ^25^, ^28^ ^]^ During the following maturation process, early endosomes fuse to form late endosomes, leading to invagination of the endosomal membrane into the lumen and form intraluminal vesicles (ILVs). This in turn leads to the formation of multivesicular endosomes or MVBs with a characteristic multivesicular appearance. MVBs ultimately fuse with the plasma membrane and release the ILVs into the extracellular milieu in the form of exosomes. Alternatively, MVBs can fuse with lysosomes/autophagosomes to be degraded.^[^ ^5^, ^25^ ^]^ Briefly, the generation of exosomes consists of three steps: biogenesis, transport and release. Microvesicles (MVs) are derived from the plasma membrane by direct outward budding and fission, which is reminiscent of a reverse version of endocytosis.^[^ ^5^, ^27^ ^]^

### 2.1 MVB Biogenesis

The endosome sorting complexes required for transport (ESCRT) machinery plays an important role in the formation of MVBs and ILVs (Figure 1). The ESCRTs comprise four distinct complexes (ESCRT‐0, ‐I, ‐II, and ‐III) and the accessory Vps4 complex, with each of them consisting of several subunits (**Box** **1**) (reviewed in refs. [29, 30, 31]).

**Box 1**

The endosomal sorting complexes required for transport (ESCRT) machinery was originally identified in budding yeast through genetic and biochemical characterization of vacuolar protein sorting (vps) mutants.^[^ ^173^ ^]^ ESCRT complexes have been extensively studied in yeast and human, and comprise ESCRT‐0, ESCRT‐I, ESCRT‐II, ESCRT‐III subcomplexes and ATPase complex, as well as several accessory proteins (see Table). Among them, ESCRT‐0, ESCRT‐I, ESCRT‐II and ATPase complex are stable complexes existing before recruitment,^[^ ^38^, ^50^, ^173^, ^251^ ^]^ while ESCRT‐III subunits are dynamically assembled after recruitment.^[^ ^51^, ^53^ ^]^ The ESCRT machinery plays a vital role in a series of membrane remodeling events, including multivesicular body (MVB) biogenesis, cytokinetic abscission and viral budding.^[^ ^252^, ^253^ ^]^ The table lists the main components involved in the ESCRT pathway.^[^ ^29^, ^31^, ^252^, ^254^, ^255^ ^]^

**Table:** 

Complex	Component		Interaction site		
	*Homo sapiens*	*Saccharomyces cerevisiae*	Membrane	Intracomplex	Intercomplex
ESCRT‐0	STAM1, 2	Hse1		HRS	HD‐PTP
	HRS	Vps27	PtdIns3P	STAM	TSG101, clathrin
ESCRT‐I	TSG101 (VPS23)	Vps23		VPS28, VPS37, MVB12,	HRS, ALIX, HD‐PTP
	VPS28	Vps28		TSG101	EAP45, CHMP6
	VPS37A, B, C, D	Vps37		TSG101, MVB12	
	MVB12A, B^a)^	Mvb12		TSG101, VPS37	
	UBAP1^a)^				HD‐PTP
ESCRT‐II	EAP30 (VPS22)	Vps22		EAP20, EAP45	
	EAP20 (VPS25)	Vps25		EAP30, EAP45	CHMP6
	EAP45 (VPS36)	Vps36	PtdIns3P	EAP30, EAP20	VPS28
ESCRT‐III	CHMP2A, B (VPS2A, B)	Vps2		CHMP1, 3	VPS4, LIP5
	CHMP3 (VPS24)	Vps24	PtdIns3P	CHMP2, 4	VPS4
	CHMP4A, B, C (SNF7A, B, C)	Vps32 (Snf7)		CHMP3, 6, 7	VPS4, ALIX
	CHMP6 (VPS20)	Vps20	Myristoylation	CHMP4	VPS4, EAP20, VPS28, HD‐PTP
	CHMP1A, B (DID2)	Vps46 (Did2)		CHMP2	VPS4, LIP5
	CHMP5 (VPS60)	Vps60			VPS4, LIP5
	CHMP7			CHMP4	
ATPase	VPS4A, B (SKD1)	Vps4		LIP	CHMP1‐6
	LIP5	Vta1		VPS4	CHMP1, 2, 5
Accessory	ALIX	Bro1	LBPA		TSG101, CHMP4
	HD‐PTP				UBPA1, TSG101, CHMP4, STAM

Abbreviations: STAM, signal transducing adaptor molecule; Vps, vacuolar protein sorting; HRS, hepatocyte growth factor‐regulated tyrosine kinase substrate; Hse1, heat shock element 1; TSG101, tumor susceptibility gene 101 protein; MVB12, multivesicular body subunit 12; UBAP1, ubiquitin associated protein 1; CHMP, charged multivesicular body protein; SKD1, suppressor of K^+^ transport defect 1; LIP5, LYST‐interacting protein 5; Vta1, vesicle trafficking 1; ALIX, ALG‐2 interacting protein X; HD‐PTP, His domain protein tyrosine phosphatase; PtdIns3P, phosphatidylinositol 3‐phosphate; LBPA, lysobisphosphatidic acid.

^a)^ MVB12A, B and UBAP1 are mutually exclusive.

**Figure 2:**
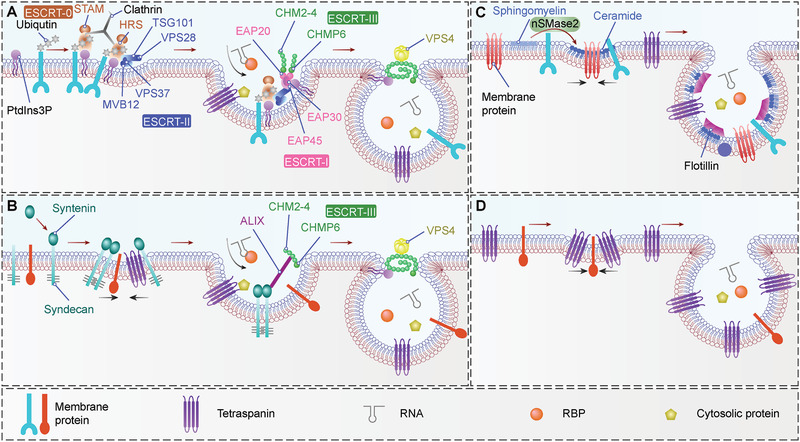
MVB biogenesis machineries. Multiple molecular mechanisms of ILV generation in MVB have been revealed. **A)** In the canonical ESCRT‐dependent pathway, ubiquitinated proteins in the endosomal membrane are recognized by ESCRT‐0, which is recruited to the endosomal membrane by PtdIns3P binding and subsequently clustered into microdomains via clathrin binding. Then ESCRT‐0 recruits ESCRT‐I, and ESCRT‐I recruits ESCRT‐II. ESCRT‐I and ESCRT‐II coordinately induce the budding of the endosomal membrane and confine cargos within the buds. ESCRT‐III components are dynamically recruited for membrane scission of the ILV necks and disassembled after ILV scission via VPS4. In a non‐canonical ESCRT‐dependent pathway, HD‐PTP binds to ESCRT‐0 and coordinately recruits ESCRT‐I and ESCRT‐III, bypassing the need for ESCRT‐II. **B)** In the syndecan‐syntenin‐ALIX pathway, membrane budding and cargo clustering can occur independently of ubiquitin and ESCRT‐0, but ESCRT‐III and VPS4 are required for the scission step. **C)** Ceramide, generated from sphingomyelin by mSMase2, plays a key role in the ESCRT‐independent pathway of ILV biogenesis. Ceramide can form lipid raft microdomains, which might trigger the conversion of ILVs into MVBs. **D)** CD63 plays a vital role in the ESCRT‐independent pathway of ILV biogenesis. CD63 can form tetraspanin‐enriched microdomains, which might trigger the conversion of ILVs into MVBs. Abbreviations: MVB, multivesicular body; ILV, intraluminal vesicle; ESCRT, endosome sorting complex required for transport; PdtIns3P, phosphatidylinositol 3‐phosphate; STAM, signal transducing adaptor molecule; HRS, hepatocyte growth factor‐regulated tyrosine kinase substrate; TSG101, tumor susceptibility gene 101 protein; VPS, vacuolar protein sorting; MVB12, multivesicular body subunit 12; CHMP, charged multivesicular body protein; HD‐PTP, His domain protein tyrosine phosphatase; ALIX, ALG‐2 interacting protein X; nSMase2, neutral sphingomyelinase 2.

In the canonical ESCRT‐dependent pathway of ILVs (**Figure** **2**A), the ESCRT complexes are recruited to the endosomal membrane in a stepwise manner. First, phosphatidylinositol‐3‐phosphate (PtdIns3P), an abundant phosphoinositide in endosomal membranes,^[^ ^32^, ^33^ ^]^ recruits the ESCRT‐0 complex to early endosomes via its HRS (Vps27 in yeast) subunit.^[^ ^34^ ^]^ Subsequently, HRS recruits clathrin,^[^ ^35^ ^]^ which induces clustering of HRS to restricted microdomains.^[^ ^36^ ^]^ HRS also binds to ubiquitin, which is essential for efficient sorting of ubiquitinated proteins into clathrin‐coated microdomains.^[^ ^37^, ^38^ ^]^ Then, ESCRT‐0 recruits ESCRT‐I to endosomal membranes via direct interaction of HRS with the TSG101 (Vps23 in yeast) subunit of ESCRT‐I.^[^ ^39^, ^40^, ^41^ ^]^ ESCRT‐I recruitment is blocked in the absence of ESCRT‐0.^[^ ^39^, ^40^, ^41^ ^]^ Indeed, TSG101 also contains a ubiquitin‐binding domain.^[^ ^42^, ^44^, ^45^ ^]^ In yeast, Vps23 (TSG101 in human) can cooperate with Vps27 (HRS in human) to increase the sorting efficiency of ubiquitinated cargos.^[^ ^46^ ^]^ Besides interacting with ESCRT‐0, ESCRT‐I also interacts with the ESCRT‐II complex. In yeast, this link is achieved by the Vps28 and Vps36 subunits of ESCRT‐I and ESCRT‐II, respectively.^[^ ^47^, ^48^ ^]^ The function of ESCRT‐I and ESCRT‐II is thought to be responsible for endosomal membrane invagination.^[^ ^49^ ^]^ ESCRT‐III assembly is initiated by ESCRT‐II recruitment,^[^ ^50^, ^51^ ^]^ which is mediated by the direct binding of Vps20 (CHMP6 in human) to Vps25 (EAP20 in human).^[^ ^52^ ^]^ ESCRT‐III recruitment is required for ILV scission into the MVB lumen.^[^ ^53^, ^54^, ^55^ ^]^ After scission, ESCRT‐III is disassociated from the membrane and recycled for additional rounds of budding. This dissociation process requires interaction with the AAA‐ATPase Vps4.^[^ ^51^, ^54^, ^55^, ^56^, ^57^, ^58^ ^]^ Vps24 might also cooperatively drive membrane scission.^[^ ^59^, ^60^, ^61^ ^]^

There are other parallel ways to recruit ESCRTs for ILV biogenesis and cargo sorting, and these are termed non‐canonical ESCRT‐dependent pathways (Figure 1). Two non‐canonical pathways have been identified in yeast, and four in mammals. In yeast, **1)** Bro1 functions as a ubiquitin receptor and works in the ILV biogenesis pathway in parallel with ESCRT‐0.^[^ ^62^ ^]^ **2)** Bro1 provides a direct bridge between ESCRT‐0 and ESCRT‐III independently of ESCRT‐I and ESCRT‐II.^[^ ^63^, ^64^ ^]^ In mammalian cells, **1)** ALIX (Bro1 in yeast) participates in the syndecan‐syntenin‐ALIX ILV biogenesis and cargo‐sorting pathway, which is independent of ubiquitination and ESCRT‐0, but dependent on ESCRT‐III^[^ ^65^ ^]^ (Figure 2B). In this mechanism, the transmembrane protein syndecan recruits syntenin, and then syntenin interacts with ALIX, which supports the biogenesis of ILVs as well as cargo sorting.^[^ ^65^ ^]^ Furthermore, the syndecan‐syntenin‐ALIX axis is modulated by heparinase,^[^ ^66^ ^]^ the small GTPase ADP ribosylation factor 6 (ARF6), and phospholipase D2 (PLD2),^[^ ^67^ ^]^ as well as the cytosolic tyrosine kinase SRC, which is upstream of ARF6 and PLD2.^[^ ^68^ ^]^ **2)** ALIX directly binds protease‐activated receptor 1 (PAR1) and recruits ESCRT‐III in an MVB biogenesis and cargo‐sorting pathway that is independent of ubiquitination, and ESCRT‐0, ‐I, and ‐II.^[^ ^69^ ^]^ Also, the adaptor protein complex‐3 (AP3) facilitates PAR1 sorting to ILVs.^[^ ^70^ ^]^ **3)** ALIX can directly recruit ESCRT‐III onto late endosomes by binding to lysobisphosphatidic acid (LBPA)^[^ ^71^ ^]^ without requiring ESCRT‐0, ‐I, and ‐II; this pathway might play a direct role in ILV formation in late endosomes and tetraspanin sorting.^[^ ^72^, ^73^ ^]^ **4)** Another Bro1 domain‐containing protein, HD‐PTP, bypasses the requirement for ESCRT‐II in ILV biogenesis and functions as a scaffold that consecutively recruits ESCRT‐0, ‐I, and ‐III.^[^ ^74^, ^75^, ^76^, ^77^, ^78^ ^]^ Specifically, HD‐PTP recruits ESCRT‐I through interacting with ubiquitin‐associated protein 1 (UBAP1), instead of MVB12.^[^ ^75^, ^76^ ^]^ Up to the present, all non‐canonical ESCRT‐dependent pathways require the engagement of ESCRT‐III and Vps24, whose functions have been discussed above.

Recent evidence has revealed that ESCRT‐independent mechanisms also participate in ILV formation, because simultaneous inhibition of ESCRT complexes does not abolish MVB formation.^[^ ^79^ ^]^ In oligodendroglial cells, ceramide is formed after hydrolytic removal of the phosphocholine moiety of sphingomyelin by neutral sphingomyelinase 2 (nSMase 2), and was proposed to participate in ESCRT‐independent ILV formation based on the finding that ceramide induces the formation of ILVs in liposomes in vitro^[^ ^80^ ^]^ (Figure 2C). This EV biogenesis pathway has been further unraveled in many cancer cells, in which EV release was decreased upon inhibition of ceramide synthesis ^[^ ^81^, ^82^, ^83^, ^84^, ^85^ ^]^ and increased upon stimulation with C6 ceramide.^[^ ^83^ ^]^ Another ESCRT‐independent pathway might be initiated by tetraspanins without the requirement of ubiquitination, ESCRT or ceramide ^[^ ^86^, ^87^ ^]^ (Figure 2D). In this mechanism, the tetraspanin CD63 is required for ILV formation and subsequent EV release,^[^ ^86^, ^88^ ^]^ whereas depletion of ESCRT or ceramide does not impair EV secretion or cargo sorting.^[^ ^87^ ^]^ More recently, Wei et al. screened a library of constitutively active forms of RAB GTPases, and found that RAB31 controlled an ESCRT‐ and tetraspanin‐independent, but ceramide‐dependent ILV formation pathway.^[^ ^89^, ^90^ ^]^

As mentioned above, MVBs can fuse either with lysosomes for degradation of their contents or with plasma membrane for EV release^[^ ^5^, ^25^ ^]^ (Figure 1). The mechanisms that determine the fate of MVBs are still largely unknown, but ISGylation of TSG101 ^[^ ^91^ ^]^ and tetraspanin 6^[^ ^92^ ^]^ have been shown to negatively regulate the release of EVs. However, the reports on the effects of tetraspanin 6 on EV release are discrepant,^[^ ^92^, ^93^ ^]^ possibly due to the differences of cell types and contexts. Similarly, there is a balance between EV secretion and macroautophagy; in the latter pathway, the autophagosomes subsequently fuse with lysosomes, resulting in degradation of their contents^[^ ^5^, ^25^ ^]^ (Figure 1). For example, inhibiting the fusion of MVBs with autophagosomes promotes the secretion of EVs.^[^ ^94^, ^95^ ^]^ Moreover, it has recently been found that expression of several autophagy‐related genes (Atg), including ATG3, ATG5, ATG7, ATG12, and LC3 (short for MAP1LC3B, microtubule‐associated 1A/1B light chain 3B protein), promotes the biogenesis of MVBs and subsequent EV release independently of canonical macroautophagy.^[^ ^96^, ^97^, ^98^ ^]^

### 2.2 MVB Transport

The transport of MVBs to the plasma membrane involves their interaction with the cytoskeleton, molecular motor and small GTPases,^[^ ^5^, ^28^, ^99^ ^]^ and shares similar mechanisms to those of other intracellular vesicles.^[^ ^100^ ^]^ The involvement of the cytoskeleton (microtubules and actin) in MVB transport is supported by the observation of oriented secretion of EVs in T cells and invasive cancer cells.^[^ ^82^, ^101^ ^]^ The unidirectional transfer of EVs from T cells to antigen‐presenting cells implies MVB trafficking along the network of microtubules in immunological synapses.^[^ ^101^, ^102^ ^]^ The molecular motors involved in this process have not been identified, though actin was shown to provide docking sites for the intracellular trafficking of MVBs in cancer cells.^[^ ^82^ ^]^ Moreover, knockdown or overexpression of the actin cytoskeletal regulatory protein cortactin increases or decreases EV secretion, respectively.^[^ ^103^ ^]^

RAB GTPases are molecular switches that regulate intracellular vesicle transport,^[^ ^104^ ^]^ including EV secretion (Figure 1). RAB11 was the first RAB GTPase shown to be involved in EV secretion.^[^ ^105^ ^]^ Overexpression of a dominant‐negative RAB11 mutant decreases the release of transferrin receptor (TfR) and heat shock cognate 71 kDa protein (Hsc70)‐containing EVs in human leukemia cell line K562.^[^ ^105^, ^106^ ^]^ Later, two different screening strategies identified several new RABs with roles in EV secretion,^[^ ^107^, ^108^ ^]^ and small hairpin RNA (shRNA)‐based screening established that RAB2B, RAB5A, RAB9A, RAB27A, and RAB27B are required for EV release in HeLa cells.^[^ ^107^ ^]^ Furthermore, silencing of RAB27A decreased EV secretion in various tumor cell lines.^[^ ^82^, ^109^, ^110^ ^]^ In parallel, by using a library of GTPase‐activating proteins, RAB35 was identified as a regulator of EV secretion in a murine oligodendroglial cell line (Oli‐neu cells).^[^ ^108^ ^]^ Inhibition of RAB35 decreased the secretion of proteolipid protein (PLP)‐bearing EVs in Oli‐neu cells^[^ ^108^ ^]^ and also in primary oligodendrocytes.^[^ ^111^ ^]^ These RABs were proposed to function in the docking of MVBs to the plasma membrane, as exemplified by RAB27 and RAB35.^[^ ^107^, ^108^ ^]^ The last RAB protein proven to function in EV release is RAB7, whose depletion severely reduced the secretion of syndecan‐syntenin‐ALIX EVs.^[^ ^65^ ^]^ In *Caenorhabditis elegans*, the Ras‐related GTPase homolog, Ral‐1 is involved in both MVB formation and fusion with the plasma membrane.^[^ ^112^ ^]^ Its mammalian homologs RalA and RalB are also both required for the secretion of exosome‐like vesicles.^[^ ^112^ ^]^

Interestingly, the regulation of EV secretion by RAB proteins is cell‐type‐dependent.^[^ ^113^ ^]^ In human HeLa cells, the depletion of RAB2B, RAB5A, RAB9A, and especially RAB27A and RAB27B, decreased the secretion of EVs, whereas inhibition of RAB11A and RAB7 did not.^[^ ^107^ ^]^ In the human RPE cell line, both RAB11 and RAB35, but not RAB27 are required for the secretion of lethal factor (LF)‐loaded EVs.^[^ ^114^ ^]^ In contrast, in *Drosophila* S2 cells, depletion of RAB11 reduced the release of wingless or evenness interrupted (Evi)‐bearing EVs, whereas neither RAB27A nor RAB35 showed a similar effect.^[^ ^115^, ^116^ ^]^ In human MCF7 breast cancer cells, RAB7 plays a role in the secretion of syntenin‐ and ALIX‐containing EVs,^[^ ^65^ ^]^ whereas it is not required in HeLa cells.^[^ ^107^ ^]^

### 2.3 Fusion of MVBs with the Plasma Membrane

The final step of exosome secretion is the fusion of MVBs to the plasma membrane, driven by soluble NSF attachment protein receptor (SNARE) proteins ^[^ ^117^, ^118^ ^]^ (Figure 1). The role of SNAREs in membrane fusion, including exocytosis, which is reminiscent of exosome secretion, has been extensively studied.^[^ ^118^, ^119^, ^120^ ^]^ The SNARE protein VAMP7, which is involved in fusion of secretory lysosomes with the plasma membrane,^[^ ^121^ ^]^ was shown to regulate the release of EVs in K562 leukemia cells,^[^ ^122^ ^]^ but inhibition of VAMP7 in MDCK cells has no effect on the secretion of EVs.^[^ ^123^ ^]^ Subsequently, two other SNAREs, syntaxin 1A (Syx1A) ^[^ ^115^ ^]^ and Ykt6,^[^ ^124^, ^125^ ^]^ were shown to be required for the secretion of EVs containing Evi and Wnt, respectively, in the tested cell types. Recently, SNAP‐23 was demonstrated to facilitate EV release in tumor cells under the control of phosphorylation mediated by pyruvate kinase type M2 (PKM2).^[^ ^126^ ^]^ In *C. elegans*, the SNARE protein Syx‐5 is involved in the fusion of MVBs to the plasma membrane, and its absence causes accumulation of MVBs beneath the plasma membrane.^[^ ^112^ ^]^

### 2.4 Biogenesis and Release of Microvesicles

The molecular mechanisms of biogenesis of microvesicles are less well characterized. Microvesicles have been referred to as ubiquitous vesicles that are formed by direct budding from the plasma membrane of healthy cells ^[^ ^1^, ^7^ ^]^ (Figure 1). One mechanism of their synthesis involves the ESCRT machinery. Knockdown of ESCRT proteins, including Alix, Tsg101, Vps22, Chmp1/3, and Vps4 reduced the secretion of Hedgehog (Hh) via EVs.^[^ ^127^ ^]^ Another mechanism involves recruitment of the ESCRT subunits TSG101 and VPS4 to the plasma membrane by adaptor protein arrestin domain‐containing protein 1 (ARRDC1), thereby promoting the generation of EVs (termed ARRDC1‐mediated microvesicles; ARMMs).^[^ ^128^ ^]^ Apart from the ESCRT machinery, small GTPase proteins, including ARF1,^[^ ^129^ ^]^ ARF6,^[^ ^130^ ^]^ and RhoA,^[^ ^131^ ^]^ also enable EVs to bud off from the plasma membrane of cancer cells. Moreover, activation of acid sphingomyelinase (A‐SMase), another member of the SMase family on the plasma membrane, can trigger the release of EVs from glial cells^[^ ^132^ ^]^ and red blood cells^[^ ^133^ ^]^ via generation of ceramide.

## 3 EV Uptake and Cell–Cell Communication

Accumulating evidence has demonstrated that EVs act as an important mediator of intercellular communication, and this has generated increasing interest in the development of EV‐based therapeutic agents. It is generally accepted that EVs exert their intercellular signaling function in two ways:^[^ ^6^, ^134^ ^]^ 1) they can transmit information to recipient cells by direct contact via their surface ligands; 2) they can transfer proteins and nucleic acids, including RNAs and DNAs to target cells.

**Figure 3:**
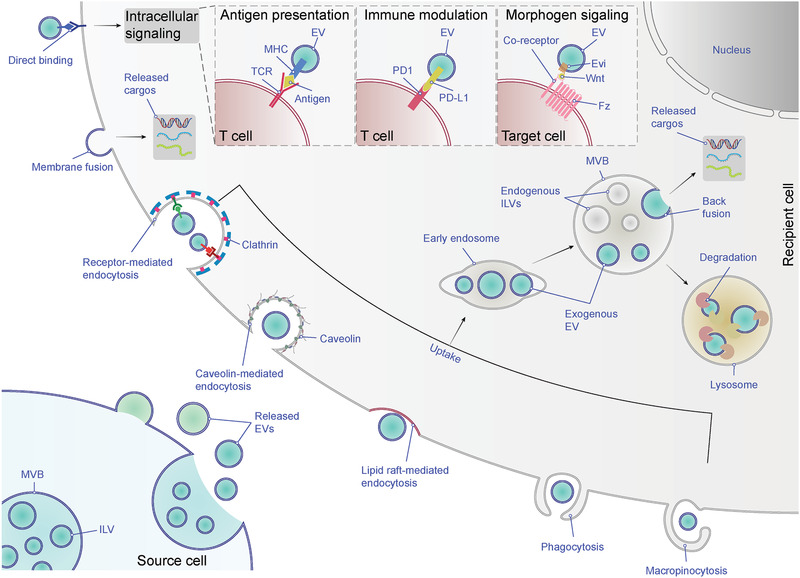
Uptake and fate of EVs. EVs can trigger intracellular signaling of recipient cells via ligand‐receptor interaction, such as antigen presentation, immune modulation and morphogen signaling. Alternatively, exogenous EVs can transfer their cargos into recipient cells by entering the cells. EVs can be internalized into recipient cells by different mechanisms, including membrane fusion and various endocytic pathways (e.g., receptor‐mediated endocytosis, caveolin‐mediated endocytosis, lipid raft‐mediated endocytosis, phagocytosis and macropinocytosis). The fusion of EVs with the plasma membrane can release their contents into the cytoplasm of recipient cells, while the endocytosed EVs reach MVBs via the canonical endosomal pathway. These internalized EVs might be degraded after the fusion of MVBs with lysosomes or be secreted from recipient cells mixed with endogenous ILVs (not shown). Also, they might back‐fuse with the limiting membrane of MVBs, leading to the release of the EV cargos into the cytoplasm, a process that is poorly understood. Abbreviations: EV, extracellular vesicle; MVB, multivesicular body; ILV, intraluminal vesicle; TCR, T‐cell receptor; MHC, major histocompatibility complex; PD1, programmed cell death protein 1; PD‐L1, programmed death‐ligand 1; Fz, Frizzled receptor.

The first mechanism depends on ligand–receptor interaction, without delivery of the EV contents into the recipient cell, and has been well studied in connection with immunomodulation (**Figure** **3**). For example, EVs derived from B lymphocytes^[^ ^18^ ^]^ and dendritic cells (DCs)^[^ ^19^, ^135^ ^]^ harbor major histocompatibility complex (MHC), and can provoke T cell‐mediated immune responses by activating cognate T cell receptors. Another example is cancer cell‐derived EVs bearing programmed death ligand‐1 (PD‐L1), which can lead to cancer immune evasion via inhibition of T cell function by binding to programmed cell death protein‐1 (PD1).^[^ ^84^, ^136^, ^137^, ^138^ ^]^ Apart from their immunomodulatory function, Wnt protein‐bearing EVs can induce Wnt signaling activity in recipient cells.^[^ ^124^, ^139^ ^]^ This property indicates that EVs could be promising candidates for developing immunomodulatory therapeutics and antitumor vaccines.

The second mechanism depends on cellular internalization or membrane fusion, leading to the entry of the EV contents into acceptor cells (Figure 3). This mechanism has been extensively investigated in various cell types and is well characterized. For instance, EVs can transfer microRNAs (miRNAs) into acceptor cells to downregulate expression of target genes^[^ ^21^, ^22^, ^101^, ^140^, ^141^ ^]^ and deliver messenger RNAs (mRNAs) to be functionally translated.^[^ ^20^, ^21^, ^22^, ^142^ ^]^ Other functionally important genetic materials, including genomic DNAs (gDNAs),^[^ ^143^, ^144^ ^]^ mitochondrial DNAs (mtDNAs),^[^ ^145^, ^146^ ^]^ and long noncoding RNAs (lncRNAs),^[^ ^143^, ^147^ ^]^ can also be delivered to recipient cells via EVs. EVs can also transfer protein cargos, such as oncoproteins from cancer cells to neighboring cells^[^ ^22^, ^109^, ^148^, ^149^, ^150^ ^]^ and deliver a retrograde signal (i.e., synaptotagmin 4) from presynaptic to postsynaptic cells.^[^ ^151^ ^]^ Nevertheless, although the cargo delivery capacity of EVs has been demonstrated and delivery systems based on EVs have already been developed,^[^ ^152^ ^]^ the mechanisms of EV uptake and cargo delivery into the cytosol of recipient cells are still poorly understood.

Given that EV–cell interactions and virus–cell interactions share topological similarities, the mechanisms of viral uptake provide useful paradigms for exploring the mechanisms of EV uptake.^[^ ^15^, ^153^ ^]^ Currently, two models of EV uptake have been proposed and are widely accepted: direct membrane fusion and endocytosis.^[^ ^154^, ^155^ ^]^ Indeed, EV uptake via direct fusion with the cell's plasma membrane has been observed directly using fluorescent lipid dequenching.^[^ ^154^ ^]^ Parolini et al. used a lipid fluorescent probe, R18, to label EVs derived from melanoma cells, and demonstrated that a part of these labeled EVs directly fused with the plasma membrane of acceptor cells.^[^ ^156^ ^]^ Subsequently, Montecalvo et al. obtained similar results in bone‐marrow‐derived DCs using the dequenching method, and further showed that EV uptake could occur via fusion of EVs with the plasma membrane.^[^ ^157^ ^]^ Nevertheless, the fusion‐based pathway is not the main uptake mechanism. Most experimental evidence indicates that EVs are mainly taken up into endosomes via endocytosis.^[^ ^157^, ^158^, ^159^, ^160^ ^]^ Endocytosis is a generic term for cellular internalization, which can be generally subdivided into five categories: receptor‐mediated endocytosis (also known as clathrin‐mediated endocytosis), caveolin‐mediated endocytosis, lipid raft‐mediated endocytosis, phagocytosis and macropinocytosis.^[^ ^154^, ^155^, ^161^ ^]^ All of these mechanisms have been observed in various cell types (reviewed in refs. [154, 155, 162]).

Here, we focus on receptor‐mediated endocytosis, because this uptake pathway is a highly specific process that can occur only when EVs and acceptor cells share the right combination of ligand and receptor. This feature offers the possibility of controlling the tropism of engineered EVs. Though EVs naturally possess a broad tropism,^[^ ^152^, ^163^ ^]^ there are certainly examples of cell‐type‐specific uptake of EVs,^[^ ^164^, ^165^ ^]^ and introducing a targeting moiety can markedly improve tropism for target cells.^[^ ^163^ ^]^ For example, Morelli et al. found that both EV tetraspanins CD9 and CD81 and cellular integrin *α*_v_/*β*_3_ contribute to EV uptake by DCs.^[^ ^158^ ^]^ Further, integrin CD11a and its ligand CD54 (also known as intercellular adhesion molecule 1, ICAM‐1) on both EVs and DCs play a role in EV uptake by DCs.^[^ ^158^ ^]^ Nazarenko et al. reported that Tspan8‐CD49 complex‐containing EVs could be selectively internalized by endothelial cells ^[^ ^166^ ^]^ in a process mediated by the ligand CD54 expressed on acceptor cells.^[^ ^167^ ^]^ Integrins *α*_6_/*β*_4_ and *α*_v_/*β*_5_ on tumor‐derived EVs ^[^ ^165^ ^]^ and scavenger receptors on endothelial cells and patrolling macrophages ^[^ ^160^ ^]^ also mediated the cell‐specific uptake of EVs. Another example of a receptor‐mediated EV uptake pathway involves heparan sulfate proteoglycans (HSPGs) expressed on recipient cells.^[^ ^164^ ^]^ In all the aforementioned studies, chemical or antibody intervention or genetic deficiency reduced the EV uptake by recipient cells.

Thus far, many studies have demonstrated that EVs can be taken up via a receptor‐mediated endocytosis pathway, which can be manipulated at least to some extent.^[^ ^24^ ^]^ However, due to the heterogeneity of EVs and the existence of plural coexisting uptake routes, there is still a long way to go to improve the tropism of EVs in order to achieve targeted delivery. Moreover, compared with direct fusion, endocytosis is a more common entry route of EVs, so the endosome/lysosome is a likely destination for EV‐delivered content.^[^ ^155^ ^]^ Nevertheless, several studies have shown that endocytosed EVs can functionally influence acceptor cells,^[^ ^159^, ^160^, ^168^ ^]^ indicating that EV‐contained cargos can escape from the degradative pathway. In these cases, release of the cargos of internalized EVs might be achieved through a back‐fusion process, in which EVs fuse with the limiting membrane of the MVB ^[^ ^169^ ^]^ (Figure 3). However, the mechanism of this putative back‐fusion process remains largely to be elucidated, and it is unclear how recipient cells discriminate EV contents to be functionally delivered or degraded.

## 4 Cargo Sorting into EVs

The ESCRT machinery plays a key role not only in the biogenesis of ILVs, but also in protein sorting, particularly for ubiquitinated cargos ^[^ ^170^ ^]^ (Figure 2A). ESCRT‐0, ‐I, ‐II have ubiquitin‐binding modules that interact directly with ubiquitinated proteins and are necessary for cargo sorting.^[^ ^171^ ^]^ Both ESCRT‐0 subunits, HRS and STAM (Vps27 and Hse1 in yeast), contain ubiquitin‐interacting motifs (UIMs), which initiate the sorting of ubiquitinated membrane proteins.^[^ ^37^, ^38^, ^172^ ^]^ After clustering ubiquitinated cargos to clathrin‐coated microdomains, ESCRT‐0 hands them over to ESCRT‐I and ‐II. Among the ESCRT‐I subunits, both TSG101 (Vps23 in yeast) and MVB12/UBAP1 contain a ubiquitin‐binding domain that binds ubiquitinated cargos, and this seems to be crucial for cargo sorting.^[^ ^75^, ^173^, ^174^ ^]^ Among the ESCRT‐II subunits, Vps36 (EAP45 in human) can bind ubiquitin,^[^ ^175^, ^176^ ^]^ as well as ESCRT‐I and lipid.^[^ ^48^, ^177^, ^178^ ^]^

In one non‐canonical ESCRT‐dependent pathway, yeast Bro1 (ALIX in humans) functions as ubiquitin receptor, which sorts ubiquitinated cargos into ILVs, bypassing the need for ESCRT‐0.^[^ ^62^ ^]^ In another mechanism, Bro1 functions as a bridge between ESCRT‐0 and ESCRT‐III in parallel to ESCRT‐I and ‐II, and is also required for ubiquitinated cargo sorting.^[^ ^64^ ^]^ In the syndecan‐syntenin‐ALIX pathway in mammalian cells, CD63 interacts with syntenin,^[^ ^179^ ^]^ and syndecan‐syntenin regulates the sorting of the tetraspanin CD63 and its EV release ^[^ ^65^ ^]^ (Figure 2B). In contrast, the sorting of tetraspanins CD9 and CD81, as well as fortillin‐1, is not affected by syndecan‐syntenin.^[^ ^66^, ^68^ ^]^ ALIX (Bro1 in yeast) can directly bind various proteins through its YPX_n_L motifs, and this drives cargo sorting in a way that is independent of ubiquitination as well as ESCRT‐0 and ‐I, but depends on ESCRT‐III.^[^ ^69^, ^180^ ^]^ Alternatively, ALIX can sort tetraspanins (e.g., CD9, CD63 and CD81) into EVs regulated by ubiquitination, but not canonical ubiquitinated cargos (e.g., EGFR), in a LBPA‐/ESCRT‐III‐dependent pathway, which does not require ESCRT‐0, ‐I, or ‐II.^[^ ^73^ ^]^ Another Bro1 domain‐containing protein, HD‐PTP can bypass the requirement for ESCRT‐II and function as a scaffold that consecutively recruits ESCRT‐0, ‐I, and ‐III.^[^ ^74^, ^75^, ^76^, ^77^, ^78^ ^]^ In this process, HD‐PTP is required for ubiquitinated cargo sorting into ILVs and for MVB morphogenesis,^[^ ^74^, ^76^ ^]^ but is dispensable for ILV formation.^[^ ^76^ ^]^

ESCRT‐III has no known ubiquitin binding ability, which is consistent with its role in vesicle budding and scission, but not in cargo sorting (Figure 2A,B). The ESCRT‐III subunit, Vps24 (CHMP3 in human) can bind specifically to PtdIns3P,^[^ ^181^ ^]^ which might serve to stabilize ESCRT‐III on membranes.

In the ESCRT‐independent pathway, cargo sorting to ILVs of MVBs does not require ubiquitination or ESCRT complexes.^[^ ^182^, ^183^ ^]^ In the ceramide‐dependent and ESCRT‐independent EV biogenesis pathway, ceramide forms a lipid raft‐like microdomain, which can sort PLP into ILVs ^[^ ^80^ ^]^ (Figure 2C). The sorting of CD63 as well as CD81 and flotillin to ILVs may be under the control of sphingosine 1‐phosphate (S1P) signaling; S1P is a metabolite of ceramide.^[^ ^184^ ^]^ Meanwhile, these tetraspanins can regulate sorting of various cargos to ILVs.^[^ ^81^, ^84^ ^]^ In the tetraspanin‐dependent and ESCRT‐independent EV biogenesis pathway, the protein PMEL is sorted to ILVs by interacting with the tetraspanin CD63 ^[^ ^86^, ^87^ ^]^ (Figure 2D). This cargo‐sorting capacity of tetrapanins is also supported by other studies, though the exact EV biogenesis pathways in which they were involved were not well defined.^[^ ^93^, ^185^, ^186^, ^187^, ^188^, ^189^ ^]^ Since tetraspanins are ubiquitous transmembrane proteins in late endosomes,^[^ ^190^ ^]^ and they can sort both ubiquitinated and non‐ubiquitinated cargos into ILVs,^[^ ^93^, ^185^, ^187^, ^188^ ^]^ they are thought to participate in various EV biogenesis pathways.

Apart from sorting of transmembrane proteins into ILVs, some cytosolic proteins can also be selectively targeted to EVs (Figure 2). For instance, Hsc70 is co‐sorted into EVs along with TfR.^[^ ^191^, ^192^ ^]^ Moreover, Hsc70 can selectively sequester cytosolic proteins containing a KFERQ‐motif into ILVs.^[^ ^193^ ^]^ Cytosolic proteins *β*‐catenin ^[^ ^81^ ^]^ and apolipoprotein E (ApoE) ^[^ ^87^ ^]^ can also be co‐sorted into EVs with other transmembrane proteins. In addition, some cytosolic proteins modified by phosphorylation^[^ ^194^ ^]^ and farnesylation^[^ ^195^ ^]^ are sorted into ILVs and released by EVs, though the mechanisms involved are unclear.

**Figure 4:**
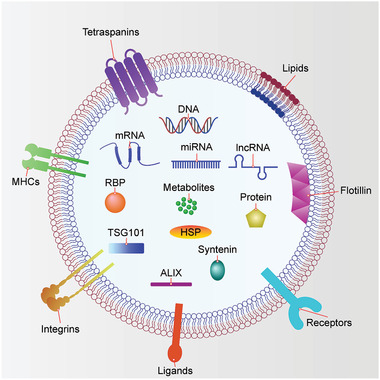
EV composition. EV contains various DNAs, RNAs, proteins, lipids, and metabolites. Some of these components are involved in the biogenesis of EVs, whereas most others are enriched in EVs during biogenesis. DNAs include dsDNA, ssDNA, and mtDNA. RNAs include mRNA, miRNA, lncRNA, tRNA, snoRNA, snRNA, Y‐RNA, vRNA, piRNA, mtRNA, and circRNA. Membrane proteins include tetraspanins (e.g., CD63, CD9, and CD81), MHC molecules, integrins, ligands (e.g., Hh, PD‐L1), receptors (e.g., EGFR, PDGFR), and flotillins. Lumen proteins include TSG101, ALIX, HSPs, syntenin, and RAB GTPases. Lipids include ceramide, phosphatidylserine, sphingomyelin, and cholesterol. Note that no single EV contains all of these components. Abbreviations: EV, extracellular vesicle; dsDNA, double‐stranded DNA, ssDNA, single‐stranded DNA; mtDNA, mitochondrial DNA; mRNA, messenger RNA; miRNA, microRNA; lncRNA, long non‐coding RNA; tRNA, transfer RNA; snoRNA, small nucleolar RNA; snRNA, small nuclear RNA; vRNA, vault RNA; piRNA, piwi‐interacting RNA; mtRNA, mitochondrial RNA; circRNA, circular RNA; MHC, major histocompatibility complex; Hh, Hedgehog; PD‐L1, programmed death‐ligand 1; EGFR, epidermal growth factor receptor; PDGFR, platelet‐derived growth factor receptor; TSG101, tumor susceptibility gene 101 protein; ALIX, ALG‐2 interacting protein X; HSP, heat shock protein; RBP, RNA binding protein.

Besides these sorted proteins, EVs also contain a variety of nucleic acids, including RNAs and DNAs ^[^ ^25^, ^196^ ^]^ (**Figure** **4**). Initially, miRNAs and mRNAs were identified in EVs.^[^ ^20^, ^21^, ^22^ ^]^ Subsequently, many other kinds of RNAs were identified in EVs,^[^ ^197^, ^198^, ^199^, ^200^, ^201^ ^]^ including transfer RNAs (tRNAs), small nucleolar RNAs (snoRNAs), small nuclear RNAs (snRNAs), Y‐RNAs, vault RNAs (vRNAs), lncRNAs,^[^ ^143^, ^147^ ^]^ piwi‐interacting RNAs (piRNAs), mitochondrial RNAs (mtRNAs) and circular RNAs (circRNAs).^[^ ^202^ ^]^ DNAs were also identified in EVs, including single‐stranded DNAs (ssDNAs),^[^ ^143^ ^]^ double‐stranded DNAs (dsDNAs) ^[^ ^203^, ^204^ ^]^ and mitochondrial DNAs (mtDNAs).^[^ ^146^ ^]^

During the process of EV biogenesis, various proteins, including RNA‐binding proteins (RBPs),^[^ ^205^ ^]^ are selectively sequestered into EVs ^[^ ^23^ ^]^ The RBPs contain a sequence‐specific RNA‐binding domain (RBD) that sorts a set of RNAs into EVs.^[^ ^147^, ^206^, ^207^, ^208^ ^]^ For example, hnRNPA2B1 recognizes the GGAG/CCCU motifs in the 3’ end of miRNAs ^[^ ^206^ ^]^ and the 5’ end of lncRNA (i.e., lncARSR),^[^ ^147^ ^]^ which cause specific miRNAs and lncARSR to be concentrated into EVs. Similarly, SYNCRIP (also known as hnRNPQ) directly binds to specific miRNAs and sorts them into EVs by recognizing a GGCU motif.^[^ ^207^, ^208^ ^]^ Also, there are RBPs that facilitate miRNA sorting into EVs, though their RBDs have not been identified.^[^ ^207^, ^209^ ^]^ Apart from RBPs, some components of the RNA‐induced silencing complex (RISC) (e.g., Ago2) are present in EVs.^[^ ^141^, ^194^, ^210^ ^]^ Overall, these proteins cause a set of miRNAs to congregate into EVs. Other mechanisms also exist, but it is not clear whether RBPs/RISC are involved.^[^ ^211^, ^212^ ^]^

## 5 EV Bioengineering

**Figure 5:**
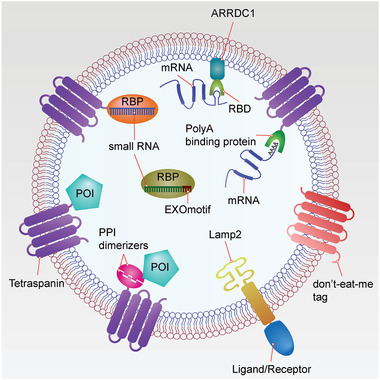
EV bioengineering. Various therapeutic nucleic acids and proteins can be incorporated into EVs through genetic engineering. Theoretically, any EV membrane protein can be used for fusion to therapeutic target moieties. To load therapeutic mRNAs into EVs, RBDs (e.g., L7Ae peptide, and Tat peptide) can be fused with EV membrane proteins (e.g., tetraspanins: CD9, CD63 or CD81, and ARRDC1), which can recruit mRNAs with their 3′UTR fused with an RBD recognition element (e.g., C/D_box_ RNA structure, and TAR element). Another strategy is to fuse a poly(A) binding protein with EV membrane proteins to selectively recruit mRNAs into EVs. To load therapeutic small RNAs (e.g., miRNA, piRNA) into EVs, RBPs (e.g., Ago, PIWI‐like protein) can be fused with EV membrane proteins to recruit them into EVs. Also, EXOmotifs can be incorporated in these small RNAs to facilitate their enrichment into EV through EV‐enriched RBPs (e.g., hnRNPA2B1, and SYNCRIP). LncRNAs can be enriched into EVs by using similar strategies. To concentrate therapeutic proteins into the lumen of EVs, a pair of PPI dimerizers can be fused with EV membrane proteins and POIs, respectively (e.g., light‐induced CRY2 and CIBN, rapamycin‐induced FRB and FKBP, and leucine zipper), through which POIs can be induced to interact with EV membrane fusion proteins by inducers. Alternatively, POIs can be directly fused to EV membrane proteins. To change the tropism of EVs, different ligands or receptors (e.g., RVG, iRGD, and EGFR nanobody) can be fused with EV surface proteins (e.g., Lamp2, PDGFR, and GPI‐anchor peptide). To protect EVs from phagocytosis, don't‐eat‐me tags (e.g., CD47, CD55, CD59, and CD200) can be expressed on EVs. Abbreviations: EV, extracellular vesicle; mRNA, messenger RNA; RBD, RNA‐binding domain; Tat, transactivator of transcription; UTR, untranslated region; ARRDC1, arrestin domain‐containing protein 1; TAR, *trans*‐activating response; miRNA, microRNA, piRNA, piwi‐interacting RNA; RBP, RNA‐binding protein; Ago, Argonaute; lncRNA, long non‐coding RNA; PPI, protein‐protein interacting; POI, protein of interest; CRY2, cryptochrome 2; CIBN, truncated version of cryptochrome‐interacting basic helix–loop–helix 1; FRB, FKBP rapamycin binding; FKBP, FK506‐binding protein; EGFR, epidermal growth factor receptor; GPI, glycosylphosphatidylinositol; PDGFR, platelet‐derived growth factor receptor.

Bioengineering of EVs employs genetic methodology to design and produce EVs with novel functionalities and properties based on knowledge of EVs biogenesis, secretion and uptake pathways (**Figure** **5**). Currently, EV engineering capabilities include cargo loading and target delivery using genetic and nongenetic methods both in vitro and in vivo (reviewed in refs. [24, 213, 214]). In this review, we focus on the bioengineering of EVs—using molecular engineering techniques to manipulate the cellular machinery for boosting production of EVs, sorting cargos into EVs in parent cells and target EVs to the desired recipient cells.

Since the finding of that EVs could horizontally transfer miRNAs and mRNAs between cells,^[^ ^20^, ^21^, ^22^ ^]^ and participate in cell–cell communication,^[^ ^6^ ^]^ EVs have attracted increasing attention. The natural origin and biological properties of EVs are particularly advantageous for application of EVs as a specific, effective and safe delivery system.^[^ ^134^ ^]^

The initial target of EV bioengineering was simply to load small RNAs into EVs, inspired by the finding that EVs can deliver RNAs.^[^ ^20^, ^21^, ^22^ ^]^ However, because the mechanisms that direct RNA sorting into EVs remained unclear, loading of small RNAs into EVs was primarily performed through cell transfection approaches. Overexpression of desired miRNAs in cells allowed these miRNAs to be incorporated into EVs and subsequently released.^[^ ^215^ ^]^ Indeed, Kosaka et al. showed that miRNAs overexpressed in HEK293 and COS‐7 cells could be released via a ceramide‐dependent pathway.^[^ ^215^ ^]^ A similar transfection‐based approach was used to load therapeutic small RNAs into EVs in other studies.^[^ ^216^, ^217^ ^]^ More recently, the molecular mechanisms behind EV RNA sorting have been largely uncovered,^[^ ^147^, ^206^, ^207^, ^209^, ^210^, ^212^, ^218^, ^219^ ^]^ which has greatly facilitated EV engineering for RNA loading. In particular, it was found that various RBPs in EVs could recognize and bind different RNAs that are enriched in EVs.^[^ ^220^ ^]^ These enriched RNAs share a common seed sequence, hereafter termed the EXOmotif, for their cognate RBPs. Loading into EVs can be enhanced by incorporating the EXOmotif into an RNA that normally would not be exported via EVs. For example, hnRNPA2B1, which is the first protein identified as being involved in EV miRNA sorting, recognizes the GGAG EXOmotif.^[^ ^206^ ^]^ Mutations in this EXOmotif reduce the EV accumulation of miR‐601, while introducing this EXOmotif into miR‐17 increases its loading into EVs.^[^ ^206^ ^]^ Another RBP SYNCRIP recognizes the GGCU EXOmotif, and insertion of this motif into miRNAs efficiently enhances the accumulation of chimera‐miRNAs into EVs.^[^ ^207^ ^]^ More recently, Reshke et al. showed that silencing RNA (siRNA) could be efficiently packaged into EVs via a pre‐miRNA backbone, which greatly reduced the therapeutic dose of siRNA.^[^ ^221^ ^]^ In their study, the pre‐miRNA backbone itself was able to facilitate siRNA loading into EVs independently of luminal argonaute (Ago), as well as other reported RBPs.^[^ ^221^ ^]^

Apart from small RNAs, proteins and mRNAs can also be loaded into EVs through the application of EV bioengineering (Figure 5). One approach uses optogenetic methodology to load proteins of interest into engineered EVs via reversible protein‐protein interaction dimerizers controlled by blue light (termed “EXPLORs”). This allows cargo proteins to be efficiently incorporated into EVs and released into the EV intraluminal space by switching the blue light on and off, respectively.^[^ ^222^ ^]^ In a more recent study, Sterzenbach et al. showed that the late‐domain (L‐domain)‐containing proteins involved in EV biogenesis could be adopted to recruit soluble proteins into EVs.^[^ ^223^ ^]^ In their study, the WW‐tagged Cre protein was efficiently loaded into EVs through the L‐domain‐containing protein Ndfip1,^[^ ^223^ ^]^ which interacts with the WW domain of the Nedd4 family ubiquitin ligases.^[^ ^224^ ^]^

Bioengineering has also been employed to change the targeting properties of EVs by manipulating their membrane proteins (Figure 5). In a pioneering study by Alvarez‐Erviti et al.,^[^ ^225^ ^]^ a chimeric protein consisting of the rabies viral glycoprotein (RVG) fused with the EV membrane protein Lamp2b was expressed in the parental cells. The produced EVs containing the targeting peptide facilitated brain‐targeted delivery of siRNA for Alzheimer's disease therapy in mice. The RVG‐directed EVs were also used to treat ischemia in a monkey model after being loaded with miR‐124.^[^ ^226^ ^]^ In another study, EVs were engineered to enhance tumor targeting by fusing the Lamp2b protein to *α*v integrin‐specific iRGD peptide. When loaded with exogenous doxorubicin, these tumor‐targeted EVs could efficiently inhibit tumor growth without overt toxicity.^[^ ^227^ ^]^ Besides Lamp2b, any other protein taking part in EV biogenesis and finally retained on the EV membrane could in principle be genetically fused to targeting ligands or receptors to facilitate the tissue‐specific targeting of EVs. For instance, by fusing the GE11 peptide to the transmembrane domain of platelet‐derived growth factor receptor (PDGFR), Ohno et al. genetically engineered EVs to achieve targeted delivery of let‐7a miRNA to epidermal growth factor receptor (EGFR)‐expressing xenograft breast cancer tissue in *RAG2^−/−^* mice.^[^ ^228^ ^]^ To engineer EVs for EGFR‐expressing tumor targeting, Kooijmans et al. expressed a chimeric protein in which an anti‐EGFR nanobody is fused to glycosylphosphatidylinositol (GPI) anchor peptide at the EV membrane.^[^ ^229^ ^]^ More recently, Cheng et al. showed that EVs engineered to express EGFR and CD3 antibodies (termed “SMART‐Exos”) could induce cross‐linking of T cells and EGFR‐expressing breast cancer cells, thus eliciting potent antitumor immunity.^[^ ^230^ ^]^

In addition to changing the targeting properties of EVs, it is also desirable to increase the lifespan of EVs in order to improve the targeting efficacy ^[^ ^24^ ^]^ (Figure 5). Kamerkar et al.^[^ ^231^ ^]^ adopted this approach, based on the finding that CD47‐SIRP*α* binding inhibits phagocytosis by initiating the “don't‐eat‐me” signal;^[^ ^232^, ^233^ ^]^ consequently, the expression of CD47 on EVs (termed “iExosomes”) increases their half‐life.^[^ ^231^ ^]^ A similar effect may also be achievable by equipping engineered EVs with other don't‐eat‐me tags, such as CD200,^[^ ^24^ ^]^ CD55, and CD59.^[^ ^234^ ^]^

There have been two important approaches to engineer EVs,^[^ ^235^, ^236^ ^]^ which epitomizes state‐of‐art design principles for EV bioengineering. One approach engineered the EXOtic device, in which Kojima et al. integrated almost all the pathways of EV biogenesis, cargo sorting, secretion, targeting and delivery.^[^ ^235^ ^]^ Through overexpression of three candidate genes (STEAP3‐SDC4‐NadB) involved in EV biogenesis, the yield of EVs was increased 15‐fold to 40‐fold (depending on cell conditions). By fusing the L7Ae module to the C‐terminus of CD63, mRNAs of interest with a C/D_box_ inserted into their 3′untranslated region (3′‐UTR) could be efficiently loaded into the engineered EVs. Also, the constitutively active connexin 43 (Cx43)‐S368A mutant served as a cytosolic delivery helper to enhance transfer of RNAs from EVs to target cells. Finally, the targeting moiety (RVG‐Lamp2b) mediated delivery of the EVs to the brain. In both in vitro and in vivo Parkinson's disease (PD) models, the transplanted EXOtic device could alleviate neurotoxicity and neuroinflammation by delivering catalase mRNA.

Bioengineering has also been applied to ARMMs, a type of EV distinct from exosomes that is produced by direct plasma membrane budding.^[^ ^128^ ^]^ ARMMs can deliver active Notch receptors to recipient cells and induce non‐canonical intercellular Notch signaling.^[^ ^237^ ^]^ Wang et al. recently used ARMMs to efficiently deliver the tumor suppressor protein p53 in vivo, utilizing a chimeric protein fused to ARRDC1. Also, p53 mRNA was successfully loaded into ARMMs by fusing the transactivator of transcription (Tat) peptide to ARRDC1 and fusing its RNA binding‐motif, *trans*‐activating response (TAR) element, to p53 mRNA. In addition, WW‐Cas9 fusion protein robustly enables CRISPR‐Cas9/guide RNA complex to be sorted into ARMMs via interaction with the PPxY motifs of ARRDC1, and delivered to recipient cells.^[^ ^236^ ^]^

## 6 Conclusions and Perspectives

The processes of EV biogenesis and release are currently largely understood, and provide a rational basis for manipulating the production of EVs. Current knowledge of cargo sorting machineries also allows various therapeutic cargos, including RNAs and proteins, to be efficiently loaded into engineered EVs in genetically modified parental cells. Although EV uptake mechanisms are not yet completely understood, it is already possible to target EVs to desired target cells or tissues via engineered ligands/receptors on their surface. In this review, we have summarized the current knowledge of EV biogenesis mechanisms and bioengineering methodologies. Beyond that, there is also a flourishing field of EV engineering using nongenetic strategies in vitro (see, for example, refs. [238, 239] reviewed in ref. [240]). In addition, there is another approach using artificial nanoparticles to deliver various therapeutic cargos to desired target tissues (see, for example, refs. [241, 242, 243, 244, 245, 246]). Since these nanoparticles share many similarities with EVs, dialogue among these fields should facilitate bioengineering of EVs for therapeutic applications.

Nevertheless, our current knowledge of EV biogenesis, release, uptake and cargo sorting is still incomplete, and some pressing issues remain to be elucidated. **1)** Various ESCRT‐dependent and ‐independent pathways have been uncovered (as discussed above, and also reviewed in refs. [5, 28]), but their contributions to EV biogenesis vary markedly depending on the cell type and/or cellular state. Extensive further investigations are still needed. **2)** Some insights into the balance between exosome secretion and lysosomal degradation have emerged (see, for example, refs. [65, 91, 92, 185]), but the mechanisms that regulate this balance are still poorly understood. **3**) The loading machineries for some small RNAs and several proteins that are sorted into EVs have also been identified (discussed above), but their full scope is still largely unknown. **4)** Molecules involved in MVB trafficking have been partially uncovered in particular exosome release pathways (reviewed in refs. [5, 247]), but because they do not necessarily need to be incorporated into exosomes, many of them are still unidentified. **5)** Molecules on the EV surface certainly influence recognition and capture by acceptor cells (reviewed in refs. [6, 154]), but our knowledge of the fates of EVs and their delivered cargos remains limited. Advances in these areas will require new experimental approaches and technologies. More recently, the composition of EVs has been extensively reassessed, and some previous findings have been overturned.^[^ ^248^, ^249^ ^]^ In future, it will be necessary to interrogate EV composition at the single‐vesicle level and also to dissect EV biogenesis at the single‐cell level in order to provide a basis for proper design and engineering of EVs for therapeutic applications.

Furthermore, to apply bioengineered EVs for clinical applications, it will be essential to produce engineered EVs in clinically authorized cell types, such as human mesenchymal stem cells (MSCs), embryonic stem cells (ESCs), induced pluripotent stem cells (iPSCs), and so on. Another challenge is to scale‐up the production of EVs. Therefore, detailed characterization of EV biogenesis pathways and cargo‐sorting machineries in these cell types will be needed. Confirmation of the applicability of current bioengineering strategies to these cell types is also required, because different strategies might be required for different cell types. For instance, the EXOtic device can boost production of EVs, thus reducing the necessary scale of cell culture in principle, but it was established in HEK‐293T cells, and works with reduced efficiency in hMSCs.^[^ ^235^ ^]^ It will be important to establish whether it can be used in other stem cells. Since EVs possess natural tropism for the liver and spleen,^[^ ^163^, ^231^, ^250^ ^]^ it will also be important to alter the tropism and to improve specific targeting. The tropism of EVs for target cells and tissues has already been changed and improved through engineering the targeting ligands/receptors on the EV surface.^[^ ^163^ ^]^ For further improvement, more specific ligands/receptors will be needed, or it may be possible to integrate plural ligands/receptors on the surface of the same EV.

In conclusion, EVs are derived from cells and consequently possess excellent biocompatibility, biostability and low immunogenicity, which are highly desirable characteristics for a new therapeutic delivery system. The increasing exploration of EV biology is providing various possible strategies to design and engineer EVs for therapeutic purposes. For example, we could hijack proteins involved in cargo sorting to package various cargos, including the above‐mentioned small RNAs, mRNAs and proteins, or even DNAs in the future, into engineered EVs through genetic modifications. We could fuse proteins retained on EVs to different target ligands/receptors in order to target EVs to desired tissues. Also, we could boost the production of EVs by genetically regulating the pathways of biogenesis and secretion.

## Conflict of Interest

The authors declare no conflict of interest.
